# Antioxidant Effect of Moroccan Pomegranate (*Punica granatum* L. Sefri Variety) Extracts Rich in Punicalagin against the Oxidative Stress Process

**DOI:** 10.3390/foods10092219

**Published:** 2021-09-18

**Authors:** Lamiae Benchagra, Hicham Berrougui, Mohamed Obaidul Islam, Mhamed Ramchoun, Samira Boulbaroud, Abdelouahed Hajjaji, Tamas Fulop, Gianna Ferretti, Abdelouahed Khalil

**Affiliations:** 1 Department of Biology, Polydisciplinary Faculty, University Sultan Moulay Slimane, Beni Mellal 23020, Morocco; benchagralamiae@gmail.com (L.B.); hichamberg@gmail.com (H.B.); ramchoun_10@yahoo.fr (M.R.); sboulbaroud@gmail.com (S.B.); abhajjaji@yahoo.fr (A.H.); 2 Department of Medicine, Geriatrics Service, Faculty of Medicine and Biological Sciences, University of Sherbrooke, Sherbrooke, QC J1H 4N4, Canada; m.o.islam@pm.univpm.it (M.O.I.); Tamas.Fulop@USherbrooke.ca (T.F.); 3 Department of Clinical Science and Odontostomatology (DISCO), Polytechnic University of Marche, I-60126 Ancona, Italy; g.ferretti@univpm.it

**Keywords:** *Punica granatum* L., antioxidant activity, low density lipoprotein (LDL), J82 human bladder cell line, paraoxonase 1

## Abstract

Natural antioxidants products are widely distributed in food and medicinal plants. These natural antioxidants, especially polyphenols, exhibit a wide range of biological activities including anti-cancer, anti-inflammatory, and anti-atherosclerosis activities. Pomegranate (*Punica granatum* L.) is a rich source of polyphenolic components. The purpose of this study was to characterize the phenolic composition and flavonoids and anthocyanin content of different parts (peel and aril) of the *Sefri* variety of pomegranate. Our results showed that Peel extract was richer in these compounds than that of the Arils, especially in Punicalagin (A and B). DPPH free radical scavenging, reducing power (FRAP), β-carotene bleaching, and hydrogen peroxide scavenging assays revealed a greater dose-dependent activity of pomegranate peel phenolic extract (PPPE) compared to pomegranate aril phenolic extract (PAPE). PPPE was also more potent than PAPE concerning its ability to inhibit conjugated diene formation and to reduce α-tocopherol disappearance induced by CuSO4-mediated LDL peroxidation. Interestingly, both extracts (PPPE and PAPE) significantly inhibited lipid peroxidation and the formation of reactive oxygen species (ROS) in stressed J82 human bladder cancer cells. These results reflect the protective effects that this Moroccan variety of pomegranate can provide against the development of metabolic disorder, cancer, atherosclerosis, and cardiovascular disease. Given these properties, further studies should be undertaken to investigate possible applications of *Sefri* pomegranate extracts in the fields of food preservation and health supplements.

## 1. Introduction

Pomegranate peel, seeds, juice, and arils are a rich source of several valuable bioactive compounds with considerable nutritional, antioxidant, and other beneficial properties [1,2,3]. Peel possesses a higher polyphenol content than seeds and juice [4]. These polyphenols include punicalagin, which exhibits high antioxidant activity. Pomegranate also contains other polyphenols, including anthocyanins (delphinidin, cyanidin, and pelargonidin 3-glucosides and 3,5-glucosides) as well as flavonols [5]. Pomegranate peel is known for its healing properties with respect to inflammatory diseases, diabetes, atherosclerosis, oxidative stress, cancer, and microbial infections [6,7,8,9,10,11]. Moreover, pomegranate fruit is used in the food industry such as dairy products, charcuterie and juice preparation, and conservation. Sweet pomegranates are consumed fresh while sour pomegranates with hard seeds are generally intended for processing [12]. Even if the consumption of arils leaves less waste, the fact remains that agri-food industry use of the pomegranate generates large amounts of peel-waste and by-products that are usually poorly exploited. Pomegranate peels represent 50% of total fruit weight and are a potential source of bioactive compounds, mainly phenolic compounds with a very broad spectrum of activity [13]. Indeed, several studies have reported the effects (antioxidant, anti-cancer, anti-inflammatory, lipid-lowering, anti-hypertensive) of phenolic extracts from the peel of the pomegranate, which makes them suitable as natural ingredients [13,14,15,16,17]. In addition, the natural product supplementation industry is increasingly interested in peel waste, manufacturing capsules containing concentrated phenolic compounds based on peels or whole fruit. Moreover, pomegranate peels are also used for their dyeing properties [18]. In fact, pomegranate peels may also be used as green antimicrobial agents to reduce inorganic nanoparticle consumption on wool yarns [19]. Pomegranate waste (peels and seeds) is also used in meat preparation and conservation to prevent bacterial development and the oxidation process [17,20,21].

Morocco’s annual pomegranate production exceeds 58,000 tones from a total area of 5000 hectares [22,23] and half of this production is grown in central Morocco on the planes of the Middle Atlas (Beni Mellal-Khenifra area) and is mainly represented by the Sefri variety. There are many pomegranate varieties in Morocco. P. granatum also has considerable synonymy, in which the same genotype is known by different names in different regions.

In this study we investigated several beneficial health properties of various bioactive compounds from *Sefri* pomegranates, notably the anti-oxidative and physicochemical properties of pomegranate peel phenolic extract (PPPE) and pomegranate aril phenolic extract (PAPE) and evaluated their effects on atherosclerosis and bladder cancer cells.

LDL oxidation is considered to be a hallmark of early atherogenesis. Nutritional antioxidants such as phenolic compounds can markedly inhibit LDL oxidative damage by reducing free radicals generated during oxidative metabolism, preserving endogenous antioxidants in LDL (vitamin E and carotenoids), chelating transition metal ions, and modulating the oxidative state of the arterial cell wall. These properties act to inhibit cell-mediated oxidation of LDL and increase serum paraoxonase (PON1) activity [24].

Polyphenols and/or their derivatives are used to treat cancer. Cancer initiation may be modulated by an increase in ROS levels, which can damage DNA and stimulate pro-oncogenic signaling [25]. Oxidative stress regulates the progression of different types of cancer, including breast, liver, lung, colon, prostate, and bladder cancer.

In the Beni Mellal-Khenifra area, all parts of the pomegranate, especially peel, are used for health remedies to treat diarrhea, ulcers, nasal bleeding, and inflammation. Moreover, this fruit is highly consumed by patients with vascular disorders. This variety may have a more powerful health potential regard to its possible richness in phenolic compounds.

To the best of our knowledge, and even though various pomegranates are cultivated in different regions of Morocco, only a few studies have focused on the chemical composition and properties of the Sefri variety of pomegranate and even fewer on its biological properties, particularly in the prevention of diseases linked to oxidative stress such as cardiovascular disease and cancer. From this perspective, we conducted this study firstly to analyze the chemical composition of phenolic compounds from peels and arils of this variety. At the biological level, we took a particular interest in studying the relationship between antioxidant activity of the Sefri cultivar and its impact in preventing lipid peroxidation in human LDLs [26] and in modulating PON 1 activity and enzyme expression. On the other hand, since oxidative stress plays an important role in cancer development and progression [27], we evaluated the effect of sefri pomegranate polyphenols on reactive oxygen species (ROS) production in J82 human bladder cancer cells.

## 2. Materials and Methods

### 2.1. Chemicals

All chemicals were of analytical reagent grade and were purchased from Sigma-Aldrich Chemical Co. (Saint Louis, MO, USA), except for Trolox, FeCl_2_-4H_2_O, ethylene-di-amine-tetra-acetic acid (EDTA), butylated hydroxytoluene (BHT), ferrozine ascorbate, H_2_O_2_, potassium ferricyanide, phosphate buffer, ferric chloride, gallic acid, catechin, Folin-Ciocalteu reagent, sodium nitrite, aluminium chloride, glacial acetic acid, acetonitrile, and formic acid, which were obtained from Sigma-Aldrich Chemical Co. (Pool, UK). Ethanol, methanol, trichloroacetic acid (TCA), sodium carbonate, and sodium hydroxide were purchased from Fisher Scientific (Loughborough, UK). Synergi 4 μm Hydro-RP 80A columns (250 mm × 4.6 mm × 5 μm) and C18 5 μm columns (250 mm × 3.0 mm) were purchased from Phenomenex (Torrance, CA, USA).

### 2.2. Plant Materials

Pomegranate fruit was harvested from Sefri pomegranate trees (central Morocco; Latitude: 23°50′05″ E; Longitude: 6°48′98″ N). The authenticity of the variety was confirmed by Dr Abbas Younes, taxonomist, and a voucher specimen was conserved for further reference at our laboratory herbarium (Beni Mellal, Morocco). The fruit was washed and hand peeled. Arils were squeezed using a commercial blender to obtain pomegranate molasses. Air-dried pomegranate peels were ground to a fine powder, which was stored in a freezer.

### 2.3. Extraction of Pomegranate Phenolic Compounds

Peel powder and pomegranate aril molasses were macerated by sonication in 70% methanol/0.1% acetic acid for 48 h in the dark. The hydroalcoholic extracts were centrifuged for 10 min, and the solids were removed by vacuum filtration through a Whatman filter Grade GF 10. The supernatants were concentrated under vacuum and were then freeze-dried and stored at −80 °C until used.

### 2.4. Quantification of Total Phenolic Content

Total phenolic content (TPC) was determined using the modified Folin-Ciocalteu method [28]. Gallic acid was used as a standard equivalent. The reaction mixtures were prepared by mixing 100 µL of each sample with 500 µL of Folin-Ciocalteu reagent (1/10). The mixtures were incubated for 10 min, following which 400 µL of 20% *w*/*v* Na_2_CO_3_ was added. Following a 2-h incubation at room temperature in the dark, the absorbance was read at 760 nm using a UV-6300PC double beam spectrophotometer (VWR, Darmstadt, Germany). TPC was determined using the gallic acid calibration curve and was expressed as milligrams of gallic acid equivalents per gram of dry matter. All samples were analyzed in triplicate.

### 2.5. Quantification of Total Flavonoid Content

Total flavonoid content (TFC) was quantified using the method described by Woisky et al., with minor modifications [29]. Briefly, 50 µL of the sample was mixed with 1.5 mL of 95% methanol, 100 µL of 2% aluminium chloride, and 350 µL of distilled water. Following a 1-h incubation at room temperature, the absorbance was measured at 420 nm. A methanolic solution of quercetin was used as a reference, and TFC was expressed as milligrams of quercetin equivalents per gram of dry matter.

### 2.6. Determination of Total Anthocyanin Content

Total anthocyanin content (TAC) was determined by the pH differential method as described by Sellappan et al. [30] using two buffer systems: potassium chloride buffer (25 mM, pH 1.0) and sodium acetate buffer (400 mM, pH 4.5). The absorbance of the buffers was read at 510 and 700 nm, respectively (1).

A = (A_510nm_ − A_700nm_) _pH 1.0_ − (A_510nm_ − A_700nm_) _pH 4.5._ (1)

TAC was calculated as cyanidin-3-glucoside equivalents (2):

(2) $$\mathrm{TAC}\;\left(\frac{\mathrm{mg}}{L}\right)=\frac{A\times \mathrm{MW}\times \mathrm{DF}\times 100}{\varepsilon }$$

MW: molecular weight of cyanidin-3-glucoside (449.2 g/mol). DF: dilution factor. ε: Molar extinction coefficient (26,900 L. cm^−1^·mol^−1^).

### 2.7. HPLC Analysis of Phenolic Compounds by UV Detection

An HPLC system composed of an autosampler (SIL-HTc), a degasser (DGU-14A), a column oven (CTO-10AS), and a diode array detector (SPD-M10A) (Shimadzu, Japan), as well as an Inertsil^®^ WP300-C18 (250 × 4.6 mm, 5 µm) column and pre-column (Canadian Life Science, ON, Canada), were used. The chromatogram was monitored at 220–400 nm), with the spectra recorded continuously throughout the elution. The following two eluents were used: A, double distilled water containing 0.2% acetic acid (pH 3.0) and B, acetonitrile. The flow rate was 1 mL/min), and the gradient was optimized as follows: 5 min (0–5%) B; 5–10 min (5–13%) B; 10–13 min (13–18%) B; 13–20 min (18%) B; 20–23 min (18–25%) B; 23–35 min (25%) B; 35–40 min (25–30%) B; 40–41 min (40–85%) B; 41–50 min (85–90%); 50–54 min (90–100%); and B; 54–60 min (100–5%) B. The samples were filtered through 0.2-mm PTFE filters, and 10 µL of the sample were injected at a stable column temperature of 30 °C. The absorption wavelengths used to detect the polyphenols ranged from 200 to 400 nm. Peak areas were quantified using a calibration curve obtained using gallic acid, α and β punicalagin, and ellagic acid as external standards. For this purpose, calibration curves were prepared for each analytical standard. Linearity, the limits of detection and quantification, are cited in Table S1 (Supplementary Data).

### 2.8. Antioxidant Activity Measurement Using the DPPH Radical Scavenging Assay

The DPPH (2,2-diphenyl-1-picrylhydrazyl) assay uses the capacity of the DPPH radical to scavenge, as a measure of the antioxidant activity that prevents lipid peroxidation. The free-radical scavenging activities of PPPE and PAPE were evaluated using the DPPH assay based on the method of Zhang et al. [31], with slight modifications. A 0.06 mM solution of DPPH in methanol was prepared daily. This solution (2 mL) was mixed with 50 µL of PPPE or PAPE (0–0.4 mg/mL). The mixture was incubated in the dark at room temperature for 30 min. The decrease in absorbance was measured at 517 nm. The percentage of inhibition was calculated using the following equation:

% Inhibition = [(A_0_ − A_S_)/A_0_] × 100 (3)

where A_0_ is the absorbance of the control reaction (containing all the reagents except the test compound) and A_S_ is the absorbance of the test compound. Ascorbic acid was used as a positive control.

The half-maximal extract concentration (IC_50_) was calculated from the plotted graph of scavenging activity against the concentrations of each extract. All experiments were performed in triplicate.

### 2.9. Hydrogen Peroxide (H_2_O_2_) Scavenging Assay

The ability of PPPE and PAPE to scavenge H_2_O_2_ was determined using the method of Ruch et al. [32]. The concentration of the H_2_O_2_ solution (40 mM) prepared in 50 mM phosphate buffer (pH 7.4) was determined by measuring the absorption at 230 nm. The absorption of the assay mixture, which contained 500 µL of different concentrations of PPPE, PAPE, or a standard ascorbic acid solution (0–200 µg/mL) together with 1 mL of H_2_O_2_ was determined after 30 min against a blank solution containing phosphate buffer without H_2_O_2_. The percentage of H_2_O_2_ scavenged was calculated using the following formula:

H_2_O_2_ scavenged (%) = [(OD_control_ − OD_test_/OD_control_)] × 100 (4)

### 2.10. Ferric Antioxidant Power (FRAP) Assay

Ferric antioxidant power was determined using the potassium ferricyanide-ferric chloride assay [33]. A 500 µL aliquot of PPPE or PAPE was mixed with 1 mL of phosphate buffer (0.2 M, pH 6.6) and 1 mL of 1% K_3_Fe(CN). The mixtures were shaken well and were incubated at 50 °C for 20 min, following which 1 mL of 10% TCA was added. They were then centrifuged at 3000 rpm for 10 min. The supernatants (1.5 mL) were mixed with 1.5 mL of distilled water and 0.1 mL of 0.1% FeCl_3_. The absorbance was read at 700 nm. Ascorbic acid was used as a reference standard. The total antioxidant activity (TAA) determined by FRAP was expressed as mg of ascorbic acid equivalents per gram of dry matter (mg AAE/g dm).

### 2.11. β-Carotene-Linoleic Acid Bleaching (BCB) Assay

The antioxidant activities of PPPE and PAPE were evaluated using the β-carotene-linoleic acid (BCB) assay as per the method of Jayaprakasha et al. [34]. β-carotene (200 µg), 20 mg of purified linoleic acid, and 200 mg of Tween 40 were mixed in 0.5 mL of chloroform. After the chloroform was removed under vacuum, the emulsion was further diluted with 40 mL of distilled water. Aliquots (4 mL) of this solution were transferred into a series of tubes containing 0.2 mL of the extract. As soon as the emulsion was added to each tube, the zero-time (t = 0 min) absorbance was measured at 490 nm. The absorbance was then measured at 15-min intervals until the color of the β-carotene disappeared in the control tubes (t = 180 min). A mixture without β-carotene served as a blank. Butylated hydroxytoluene (BHT) was used as a control. The antioxidant activity (AA) of the extracts was determined in terms of β-carotene bleaching using the following formula:

AA = [1 − (A_0_ − A_180_)/(A°_0_ − A°_180_)] × 100 (5)

where A_0_ and A°_0_ are the absorbances measured at t = 0 min of the incubation of the test sample and control, respectively, and (A_180_) and (A°_180_) are the absorbances measured in the sample and control, respectively, after a 180-min incubation. The assay was carried out in triplicate.

### 2.12. Low Density Lipoprotein Isolation

LDL (low density lipoproteins) were isolated from fasting human heparinized plasma using the method of Sattler et al. [35]. Briefly, LDLs were isolated by ultracentrifugation (543,200 g) at 15 °C using a Beckman Optima TLX ultracentrifuge equipped with a TLA-100.4 rotor. The isolated LDL were dialyzed overnight at 4 °C in sodium phosphate buffer (10^−2^ M, pH 7). Protein concentrations were measured using commercial assay kits (Bio-Rad, Mississauga, ON, Canada) according to the manufacture’s protocol and were expressed as LDL total protein concentration.

The present study was conducted according to the guidelines set out in the Declaration of Helsinki. The protocol was approved by the Ethics Committee of the Sherbrooke University Institute of Geriatrics (# 2009/19). Written informed consent was obtained from all subjects.

### 2.13. Copper-Mediated LDLs Oxidation

LDLs were oxidized using transition metal ions as oxidizing agents [36]. Briefly, 100 μg/mL of LDL were suspended in 10 mmol/L sodium phosphate buffer (pH 7) and were incubated in the presence or absence of 0.2 mg/mL of PPPE or PAPE at 37 °C and in the presence of 10 μmol/L cupric sulfates for 4 h. The oxidation reactions were stopped by cooling in an ice bath after adding 300 μmol/L of EDTA, and the resulting lipid peroxides were measured immediately.

### 2.14. Biochemical Markers of Lipid Peroxidation

#### Conjugated Diene Formation and α-Tocopherol Disappearance

Lipid peroxidation was evaluated by measuring conjugated diene formation and the disappearance of vitamin E (α-tocopherol). Oxidized LDL levels, alone or in the presence of PPPE or PAGE, were continuously monitored at 234 nm as previously described by Berrougui et al. 2006 [37], to measure conjugated diene formation.

The endogenous LDL content of vitamin E was assayed by measuring the α-tocopherol content following 2-h oxidation of LDL in the presence or absence of 0.2 mg/mL of PPPE or PAPE using reverse-phase HPLC and electrochemical (ESA Coulochem II 5010A electrochemical cell, company) and UV (at 292 nm) detection as previously described [37]. α-Tocopherol was assayed on a Sephasil peptide column (C18 5 μm ST 4.6/250) (Pharmacia Biotech, Piscataway, NJ, USA). Tocopherol acetate was used as an internal standard. The mobile phase was composed of methanol/ethanol/isopropanol (88/24/10 by volume) containing lithium perchlorate (20 μmol/L) at a flow rate of 1 mL/min.

### 2.15. Paraoxonase 1 (PON 1) Protein Expression and Activity Measurement

For intracellular staining, Fu5AH hepatic cells were fixed, permeabilized using staining Kit (abcam) and stained with anti-PON1 antibody (ab24261, 1µg/1 × 10^6^ cells) for 30 min at RT. The secondary antibody used was DyLight^®^ 488 goat anti-mouse IgG (ab96879) at 1/500 dilution for 30 min at RT. Flow cytometry data were collected on a CytoFLEX instrument (Beckman Coulter, Brea, CA, USA) and analyzed using FlowJo 10.2 software (Tree Star Inc., Ashland, OR, USA).

PON1 activity was measured in plasma samples treated or not for 2 h with 0 to 80 µg/mL of PPPE or PAPE using paraoxon as a substrate, as previously described [38]. Briefly, activity was measured by combining 50 μL of the sample with 1 mL of 100 mM Tris/HCl buffer (pH 8.0) containing 2 mM CaCl_2_ and 5.5 mM paraoxon. The rate of 4-nitrophenol release was measured at 412 nm, and enzymatic activity was calculated using a molar extinction coefficient of 17,100 M^−1^ cm^−1^. One unit of PON1 activity was defined as nM 4-nitrophenol formed per minute. Plasma was obtained from subjects who provided written informed consent before being enrolled.

### 2.16. Cell Culture

The J82 (HTB1^TM^) human bladder cancer cell line (batch # 70002468) was purchased from the American Type Culture Collection (Manassas, VA, USA) via Cederlane^®^ company (Burlington, ON, Canada). The cells were cultured in Dulbecco’s modified Eagle’s medium supplemented with 10% fetal bovine serum in a humidified 95% air/5% CO_2_ atmosphere at 37 °C. Cells were trypsinized using 0.05% EDTA-0.02% trypsin. The Fu5AH (rat hepatom cell line) were kindly provided by Dr J. Genest’s laboratory (University of McGill, Montreal, QC, Canada). Fu5AH cells were maintained in minimal essential medium containing 5% bovine serum and antibiotics [39,40].

### 2.17. Determination of Reactive Oxygen Species (ROS) in J82 Cells

J82 bladder cells were seeded into 24-well cell culture plates at a density of 2.5 × 10^4^ cells/well. Following a 24-h incubation, the medium was replaced with a fresh medium containing 0.1 or 0.2 mg/mL of PPPE or PAPE. The cells were incubated for a further 24 h. They were then washed with cold PBS. DCFH-DA (10 μmol/L) was added to each well. The cells were incubated for 45 min at 37 °C and were washed with cold PBS to remove DCFH-DA that did not enter the cells. The cells were rinsed, 100 µM TBHP (tert-butyl hydroperoxide) was added, and the cells were incubated for a further 2 h. The fluorescence was immediately recorded using a Victor multilabel plate reader (PerkinElmer, Guelph, ON, Canada), and the fluorescence intensity was quantified using excitation and emission wavelengths of 492 and 517 nm, respectively [41].

### 2.18. Determination of Thiobarbituric Acid Reactive Substances (TBARS) in J82 Cells

The assay was performed as described previously, with slight modifications [42,43]. Briefly, J82 cells were pretreated or not with 0.1 or 0.2 mg/mL of PPPE or PAPE for 24 h and then with 100 µM TBHP for 3 h. The cell culture supernatants were collected and were cleared by centrifugation (13,000× *g* for 1 min at 4 °C). The supernatants were then mixed with 200 uL of 30% TCA and 200 uL of 200 mM Tris-HCl (pH 7.4) and were incubated for 10 min at room temperature. A solution of 2 M Na_2_SO_4_ and 55 mM TBA was then added, and the supernatants were incubated for 1 h at 95 °C. The supernatants were cooled on ice for 5 min and, after adding 70% TCA was centrifuged (13,000× *g* for 1 min at 4 °C). The absorbance of the supernatants was monitored at 532 nm. Total cell protein was determined using a BCA assay. The calculation of the TBARS concentration was based on the malondialdehyde standard curve calculation. Each experiment was repeated at least in triplicate.

### 2.19. Data and Statistical Analysis

The results of the experiments were expressed as means ± standard error of the mean (SEM). Mean values were compared using an unpaired t-test or ANOVA (Dunnett’s multiple comparisons test). Statistical analyses were performed using GraphPad Prism program 8 (GraphPad Software^®^, Inc., La Jolla, CA, USA). Results were considered to be significant at *p* ˂ 0.05.

## 3. Results and Discussion

### 3.1. Total Phenolic, Flavonoid, and Anthocyanin Content

In the present study, we focused mainly on investigating bioactive compounds of pomegranate and their effects in preventing some disorders and pathologies such as bladder cancer, their cardioprotective functions, and their ability to modulate oxidative-related diseases [44,45,46]. We studied their phytochemical-related biological properties, with an emphasis on anthocyanins, phenolic acids, flavonoids, and hydrolysable tannins.

Our results showed that the levels of phenolic compounds were different in different parts of pomegranates (Table 1). PPPE had a higher TPC than PAPE (283.86 ± 17.89 vs. 166.9 ± 18.10 mg GAE/g dw, respectively, *p* < 0.05). We also found that PPPE was significantly richer in flavonoids than PAPE (185.37 ± 3.05 vs. 57.43 ± 0.41 mg QE/g dw, respectively, *p* < 0.001). However, the total anthocyanin content of PPPE was non significantly richer than that of PAPE (102.97 ± 9.19 vs. 81.26 ± 18.39 mg cy-3-glu/100 g dw, respectively).

Our results also showed that pomegranates contain high levels of phenolic compounds and anthocyanidin, especially peel and aril extracts from the Sefri variety, compared to Italian, Iranian, Turkish, Indian, and Tunisian varieties (Table S2) [47]. Russo et al. reported that six old Italian varieties, as well as the international cultivar “Wonderful,” all Gaeta varieties, are quantitatively the richest in terms of TPC in the peel. However, the Moroccan Sefri variety contains even more TPC (202.22 mg GAE/g vs. 283.86 mg GAE/g Sefri) [15]. Derakhchan et al. also investigated the TPC of peel and reported that the peel of the Natanz variety has a lower TPC than the peel of the Sefri variety (276.36 vs. 283.86 mg GAE/g Sefri) [48]. All these results, including ours, corroborate the fact that the peel extract is richer in TPC than the aril extract. The total anthocyanin content of our samples was also higher than those reported in other studies conducted with other varieties of pomegranate [49,50]. However, the difference in TPC may be influenced by variations in phytochemical composition, the cultivar studied, the extraction methods, and the experimental and environmental conditions.

### 3.2. Polyphenol HPLC Analysis

**Figure 1:**
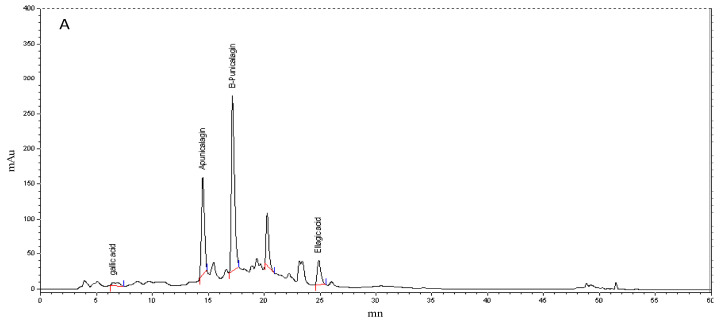

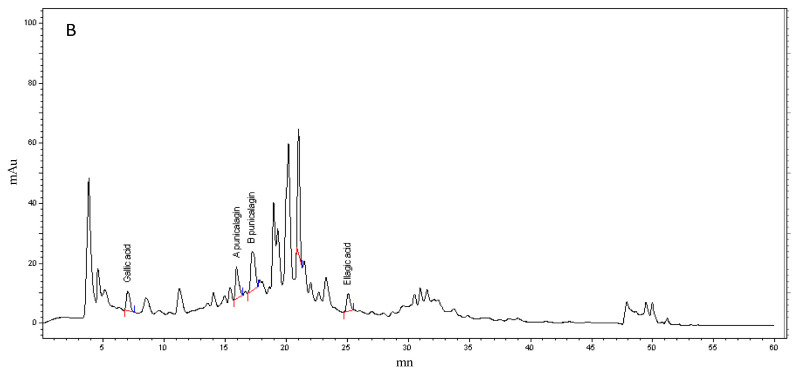
High-performance liquid chromatography-photodiode array (HPLC-PDA) chromatogram of bioactive molecules in (**A**): PPPE and (**B**): PAPE. The vertical red/blue lines correspond to the integration limits of each peak. Horizontal red lines correspond to peak detection threshold.

It is well documented that pomegranates, especially peels, are rich in ellagitannins, which can make up 66% of the total polyphenols [51]. We quantified punicalagin isomers (α and β), ellagic acid, and gallic acid by HPLC-UV and compared the results with pure standards. The chromatographic patterns of the phenolic fractions showed that α and β-punicalagin levels were higher than those of other phenolic compounds in both PPPE and PAPE (Figure 1). The retention times of gallic acid, α-punicalagin, β-punicalagin, and ellagic acid were 5.32, 14.42, 16.84, and 23.45 min, respectively. The results given in Table 2 are expressed as mg/g dry weight extract and show that the β-punicalagin content was twice as high as that of α-punicalagin (309.88 ± 13.81 mg/g dw vs. 148.95 ± 2.43 mg/g dw, respectively). These results are consistent with those obtained by Sabraoui et al. [3], who showed in a comparative study of three Moroccan pomegranate varieties that the range of punicalagin concentrations varied from 120.9 to 210.6 mg/g dw. Studies on other pomegranate cultivars also showed that the range of punicalagin concentrations varies depending on the cultivar.

### 3.3. Antioxidant Activities

DPPH radical scavenging activity was assayed using increasing concentrations of PPPE and PAPE, with ascorbate as an internal standard (0 to 0.4 mg/mL). Our results (Table 2) showed that both PPPE and PAPE exhibit significant free radical scavenging activity in a dose-dependent manner. However, PPPE exhibited 1.73-fold higher activity than PAPE, as shown by the IC_50_ values (PPPE: IC_50_ = 12.49 ± 0.60 µg/mL, PAPE: IC_50_ = 21.58 ± 4.44 µg/mL, *p* < 0.05). Ascorbic acid (IC_50_ = 6.61 µg/mL) was used as a positive control and exhibited 1.89-fold higher activity than PPPE and 3.27-fold higher activity than PAPE. The IC_50_ values obtained for DPPH in our study were much lower than those reported by Ali et al., who obtained an IC_50_ of 14.75 µg/mL for a peel extract and 128.27 µg/mL for an aril extract [52], indicating that the antioxidant effect was much greater in our study. Sabraoui et al. [3] recently reported that three Moroccan varieties had lower antioxidant activities (EC_50_ ranging from 42.71 to 65.55 µg/mL) than the Moroccan Sefri variety. However, another study conducted by Guo et al. [53], showed that a pomegranate peel extract of a Chinese variety had a high scavenging activity for hydrogen peroxide, with an IC_50_ of 0.032 µg/mL, which is much lower than our results. These differences could be explained by the growing conditions or the analytical methods used. The DPPH scavenging activity of pomegranate fruit is associated with their total phenolic, anthocyanin, and flavonoid contents, as shown in Table 1, which is why the hydro-alcoholic peel extract exhibited a higher radical scavenging activity than the aril extract. Interestingly, if TPC, especially punicalagin, gallic acid, and ellagic acid, is taken into account there is a positive correlation between TPC and the antioxidant power of PPPE, which is higher than that of PAPE [9,54]. This is in agreement with our results showing that PPPE is significantly richer in phenolic compounds than PAPE.

Our results also showed that PPPE has a better capacity to scavenge H_2_O_2_ free radicals (IC_50_ = 19.96 ± 0.02 µg/mL) than PAPE (IC_50_ = 37.06 ± 0.05 µg/mL (*p* < 0.001). In terms of antioxidant activity (AA%) at the same concentration, ascorbate exhibited the highest AA% followed by PPPE and then PAPE. At 200 ug/mL, the AA% was 98.21%, 87.8%, and 64.12%, respectively, for ascorbic acid, PPPE, and PAPE. H_2_O_2_ scavenging activity was likely affected by the concentration of phenolic compounds. Since phenolic compounds are powerful chain-breaking antioxidants, they may accelerate the decomposition of H_2_O_2_ to H_2_O and oxygen [55]. H_2_O_2_ is highly reactive and contributes to the formation of transition metal ion-dependent OH radical-mediated oxidative DNA, protein, and lipid damage [56].

The specificity and sensitivity of the DPPH and H_2_O_2_ assays did not confirm the antioxidant activities of the pomegranate extracts. Given this, FRAP and BCB assays were conducted to provide a reliable assessment of the antioxidant properties of pomegranate compounds.

Our results from the FRAP assay showed that PPPE has a higher capacity to reduce the ferric tripyridyl-triazine complex (Fe(III)-TPTZ) to a ferrous complex (Fe(II)-TPTZ) due to the electron-donating abilities of its rich phenolic compounds as expressed by TAA (374.83 ± 16.85 mg AAE/g dw) than PAPE (189.83 ± 5.29 mg AAE/g dw, *p* < 0.001) (Table 2). A similar trend was observed with the results obtained by Li et al. [57] and Sabraoui et al. [3], who found that the reducing power of a peel extract was higher than that of an aril extract. Zeljka et al. also reported that peel extracts exhibit strong antioxidant activity (100.25–176.60 μmol Trolox Eq/100 g) in reducing the Fe(III)-TPTZ complex [58]. The reducing power of pomegranate fruit parts is probably due to the action of the hydroxyl groups of phenolic compounds, which may act as electron donors. Antioxidant compounds that act as reducing agents exert their effect by donating a hydrogen atom to the ferric complex, thus breaking the radical chain reaction [57].

We evaluated the antioxidant potential of PPPE and PAPE for lipid peroxidation using the β-carotene/linoleic acid bleaching assay, which is based on a decrease in the color of β-carotene following its reaction with radicals generated when linoleic acid is oxidized. Table 2 shows that the phenolic extracts tested cause a decrease in linoleic acid oxidation. The addition of PPPE or PAPE prevented the generation of free radicals by the coupled oxidation of linoleic acid and β-carotene. At 2 mg/mL PPPE exhibited significantly higher antioxidant activity (86.83 ± 1.22%) than PAPE (55.64 ± 1.14%, *p* < 0.001). This is consistent with a study carried out by Singh et al. [59], who also reported that peel extract exhibited higher antioxidant activity (83%) than aril extract (22,6%). On the other hand, Derakhshan et al. [48] reported that a peel extract exhibited 58% antioxidant activity compared to 54% for an aril extract, while Orak et al. [60] reported that there was no significant difference in antioxidant activity between pomegranate juice (47.87%) and a peel extract (46.24%). In the β-carotene-linoleic acid system, the oxidation of linoleic acid generates radical species due to hydrogen abstraction, which occurs in the methylene groups of linoleic acid. The free radicals oxidize β-carotene by hydroperoxides. The presence of antioxidants in the extract neutralizes the linoleic free radicals as well as any other free radicals formed within the system. The oxidation of β-carotene thus depends on the antioxidant activity of the extracts [61]. Our results show clearly that PPPE and PAGE reduce the oxidation of β-carotene.

### 3.4. LDL Oxidation and Paraoxonase 1 (PON1) Activity

**Figure 2:**
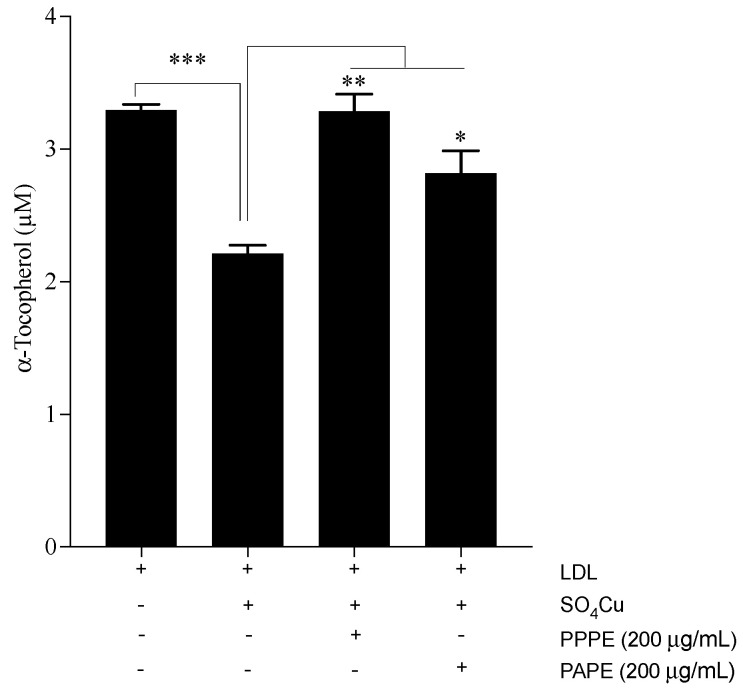
Effect of PPPE and PAPE on endogenous α-tocopherol disappearance during 4 h of CuSO_4_-induced low-density lipoprotein (LDL) oxidation. Results are expressed as the means ± sem of at least three independent assays. *** *p* < 0.001, ** *p* < 0.01 and * *p* < 0.05 indicate significant differences compared to the control.

To gain more insight into the antioxidant mechanism of pomegranate phenolic-rich extracts, we analyzed the ability of PPPE and PAPE to inhibit copper-induced LDL oxidation, prevent the disappearance of vitamin E (α-tocopherol), and promote PON1 activity. The copper-induced LDL oxidation (ox-LDL) results showed that both PPPE and PAPE significantly inhibit conjugated diene formation (*p* < 0.0001), and that PPPE was 8% more efficient than PAPE (0.154 ± 0.0017 and 0.167 ± 0.002, *p* = 0.011, respectively). This effect was confirmed by measuring the α-tocopherol content of ox-LDL in the presence or absence of PPPE or PAPE. As shown in Figure 2, the oxidation of LDL alone increased the α-tocopherol disappearance rate after 4 h of oxidation (3.29 ± 0.04 and 2.21 ± 0.06, *p* < 0.001, for non-ox-LDL and Ox-LDL, respectively). However, the phenolic pomegranate extracts significantly prevented α-tocopherol degradation. The results in Figure 2 show that PPPE exhibits a more potent effect than PAPE (3.28 ± 0.12, *p* < 0.01, and 2.81 ± 0.17, *p* < 0.05, respectively, compared to the Ox-LDL values). These results are in agreement with those obtained by Aviram et al. [62], who showed that, after 12 months of pomegranate juice consumption, oxidized serum LDL and LDL susceptibility to copper ion-induced oxidation were significantly reduced by 90% and 59%, respectively. In vitro, pomegranate peel and aril extracts exert their antioxidative activities by scavenging free radicals and inhibiting copper ion-induced LDL oxidation [63,64]. Polyphenols are significant antioxidants in pomegranate extracts. However, the differences in their antioxidative capacity can be attributed to different types of polyphenols and the polyphenol content of the various extracts. PPPE possessed a higher antioxidant capacity than PAPE. The strong antioxidant potency of PPPE may be due to its higher potential phenolic and flavonoid content as indicated by our HPLC analysis, which showed that PPPE has a higher punicalagin, gallic acid, and ellagic acid content than PAPE. These results suggest that peel polyphenols are major contributors to the antioxidative capacity of pomegranates [12,31,65].

**Figure 3:**
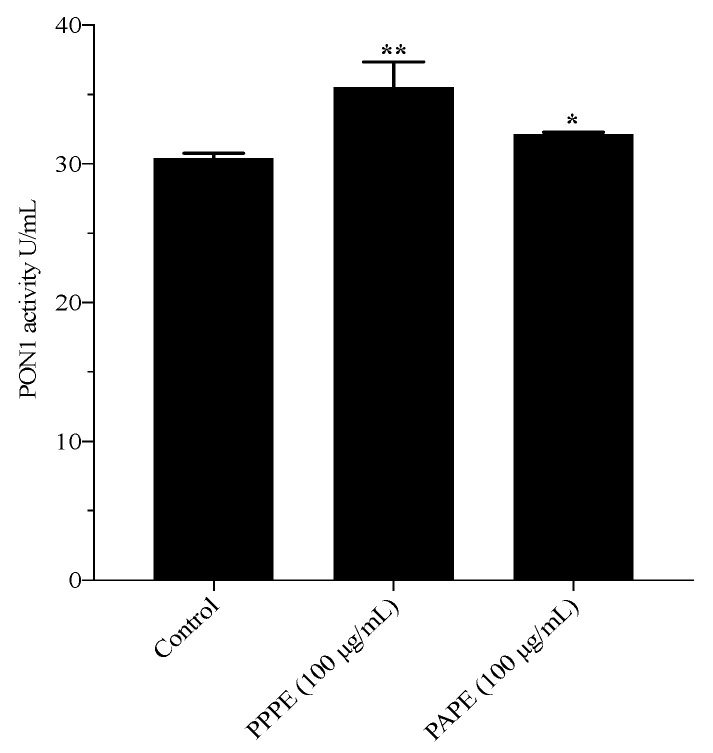
Pomegranate polyphenols improve PON1 activity. PON1 activity was measured in PPPE- or PAPE -enriched (80 µg/mL) plasma for 2 h. Results are expressed as the means ± sem of three independent assays. * *p* < 0.05, ** *p* < 0.01 and indicate significant differences compared to the control.

**Figure 4:**
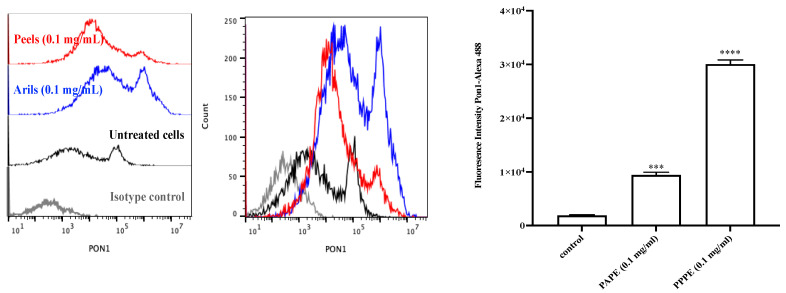
The extract of pomegranate peel and aril induces PON1 expression in Fu5AH cells. Fu5AH cells were cultured for 4 h in the presence (100 ug/mL) or absence of the extract of pomegranate’s peel or aril. The cells were washed and labelled with anti-PON1 mAbs. Expression of PON1 was determined by multi-color flow cytometry analysis in cells exposed or not to peels or arils extracts. Mean fluorescence intensities (MFI) values of FACS profiles are shown. Data are representative of three independent experiments. The asterisks indicate statistically significant differences determined by one-way ANOVA tests. *** *p* < 0.001 and **** *p* < 0.0001.

PON1 activity was evaluated in the presence of PPPE or PAPE. PON1, an HDL-associated esterase, hydrolyzes oxidized lipids, which are inactivated under oxidative stress. The incubation of human plasma with 100 µg/mL of PPPE or PAPE for 4 h at 37 °C showed that PPPE and PAPE both significantly increase plasma PON1 activity and protein expression, with PPPE exhibiting the strongest effect (Figure 3 and Figure 4, respectively). In the present study, we showed that PPPE and PAPE slightly decrease serum oxidative stress. This may be related to an increase in serum PON1 activity. PPPE was more potent (*p* ˂ 0.01) (Figure 3) than PAPE and significantly increased serum PON1 activity (*p* ˂ 0.05) as well as PON1 protein expression (*p* ˂ 0.001, *p* ˂ 0.0001; respectively) (Figure 4). These results showed that polyphenolic compounds in the pomegranate extracts, especially in peels (punicalagin, gallic acid, and ellagic acids), have a potent effect on serum PON1 activity and protein expression and are major contributors to its beneficial effects. This protection is probably the result of the ability of PON1 to hydrolyze specific oxidized lipids in oxidized lipoproteins [24]. These beneficial effects of pomegranate consumption on serum PON1 stability and activity may contribute to a delay in the development of atherosclerosis. The administration of pomegranate extracts to Apo-E deficient mice increased serum PON1 activity, with whole fruit juice being more efficient than aril juice [63]. In obese mice, daily pomegranate juice supplementation reduces oxidative stress and increases serum PON1 expression and activity [12,66]. Aviram et al. [63] showed that pomegranate juice and aril consumption resulted in a significant 43% and 22% increase in serum PON1 arylesterase activity, respectively, whereas pomegranate peel had no significant effect. They showed that pomegranate juice can preserve and enhance PON1 activity during lipoprotein oxidation. Moreover, a recent clinical study by Estrada-Luna et al. and a study using New Zealand rabbits by Dorantes-Morales et al. both showed that supplementation with an aril preparation significantly enhances PON1 activity [67,68]. In the same vein, Bentazos-Cabrera et al. reported that the consumption of fresh pomegranate juice increases PON1 activity in mice fed a high-fat diet [69]. These results indicate that there is an inverse association between serum PON1 activity and lipid peroxidation [70]. Our results suggest that pomegranate may be a source of dietary phenolic compounds that can prevent atherosclerosis and cardiovascular disease development by inhibiting lipoprotein oxidation, reducing peroxide content, and increasing PON1 activity.

### 3.5. Effect of Phenolic-Rich Pomegranate Extracts on Antioxidant Activities in J82 Cells

Cancer cells grow better in oxidative stress conditions as this increases their survival potential via various pathways that induce redox signaling activation, that in turn may lead to the suppression of tumor guardian and suppressor genes [71], the activation of survival factors such as AP-1 and NFκB, or the activation of point mutation [72]. Excess cellular ROS production may result in many harmful effects, including oxidative modifications to lipids, proteins, and DNA, that can cause various diseases.

**Figure 5:**
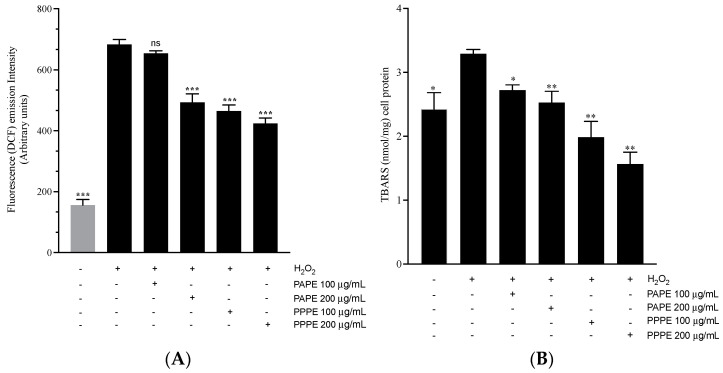
(**A**): Intracellular radical scavenging activity of PPPE and PAPE. J82 cells were treated with 100 or 200 μg/mL of PPPE or PAPE. Cells were labelled with 10 μmol/L DCFH-DA. The DCF fluorescence intensities were measured. The results are expressed as the means ± sem of more than three independent assays. *** *p* < 0.001 indicates a significant difference compared to the control. (**B**): Effects of PPPE and PAPE on TBARS levels in J82 cells. TBARS levels were assessed using a spectrophotometer. All values are expressed as the means ± sem of three independent assays. ** *p* < 0.01 and * *p* < 0.05 indicates a significant difference compared to the control (untreated cells).

In the present study, we examined the effects of PPPE and PAPE on ROS production and lipid peroxidation in J82 human bladder cancer cells. Our results showed that both PPPE and PAPE induce a significant decrease in the ROS content of J82 cells in a dose-dependent manner compared to the control (Figure 5A). PPPE (100–200 µg/mL) decreased intracellular ROS levels compared to the H_2_O_2_ control by 32.04% and 37.95%, respectively (*p* ˂ 0.001). On the other hand, 100 µg/mL of PAPE caused no significant decrease in intracellular ROS levels whereases 200 µg/mL of PAPE reduced intracellular ROS formation by 27.83% (*p* < 0.001). However, in terms of an in vitro antioxidant effect, PPPE reduced intracellular ROS formation 10% more than PAPE. These results are in agreement with those reported by Rosenblat et al., who showed that pomegranate polyphenols, especially punicalagin and gallic acid, markedly reduce ROS formation in J774 cells [73].

Lipid peroxidation, which is another approach for evaluating oxidative damage, was quantified by measuring TBARS (Figure 5B). The two methanolic extracts used in the present study both caused a decrease in lipid peroxidation compared to the control. PPPE (100 and 200 µg/mL) caused a significant reduction in MDA levels of 39.81% and 52.58% (*p* ˂ 0.01), respectively, compared to the control. PAPE (100 and 200 µg/mL) caused a much lower reduction in TBARS of 17.32% (*p* < 0.05) and 23.4% (*p* < 0.01), respectively, than PPPE. Park et al. [74] showed that a PPPE decreased ROS levels in THP-1 monocytic cells exposed to particulate matter 10-induced cytotoxicity. Zaid et al. [75] reported that a polyphenol-rich pomegranate fruit extract (POMx) decreased lipid peroxidation in human immortalized HaCaT keratinocytes following UVB-induced oxidative stress and photoaging. POMx significantly reduced peroxide accumulation, suggesting that POMx can scavenge ROS and inhibit lipid peroxidation due to its antioxidant activity. However, a study by Elango et al. [76] showed that 20 µg/mL of gallic acid (GA) isolated from pomegranate peel extracts increased ROS levels and induced the apoptosis of A549 cells through an intrinsic pathway.

## 4. Conclusions

Fruits are a rich source of vitamins, minerals, and biologically active compounds. However, they are often consumed without the peels although some fruit peels are rich in polyphenolic compounds, flavonoids, ascorbic acid, and other biologically active compounds that have a positive effect on health. Our results show that pomegranate peel and aril extracts increase serum PON1 activity, which may delay the development and progression of atherosclerosis. We also show that pomegranate extracts attenuate ROS production and lipid peroxidation in J82 human bladder cancer cells. These results indicate that pomegranate extracts from peel and arils protect against oxidative stress and exhibit anticarcinogenic activity against J82 cells. Finally, pomegranates may be a promising source of cancer-preventing agents due to their high phenolic content. However, pomegranate peel extract exhibited a better potential effect than aril extract.

## Figures and Tables

**Table 1:** Total phenolic, flavonoid, and anthocyanin content of PPPE and PAPE. Results are expressed as the mean ± sem of at least three independent assays of each sample. * *p* < 0.05 and *** *p* < 0.001 indicate significant differences compared to the control. ns: not significant.

Plant Extract	Polyphenols (mg GAE/g dw)	Flavonoids (mg QE/g dw)	Total Anthocyanin (mg cy-3-glu/100 g dw)	α-Punicalagin (mg/g dw)	β-Punicalagin (mg/g dw)	Gallic Acid (mg/g dw)	Ellagic Acid (mg/g dw)
PPPE	283.86 ± 17.89 *	185.37 ± 3.05 ***	102.97 ± 9.19, ns	148.95 ± 2.43 ***	302.38 ± 7.26 ***	5.87 ± 0.08	18.85 ± 0.41
PAPE	166.90 ± 18.10	57.43 ± 0.41	81.26 ± 18.39	40.40 ± 2.67	3.03 ± 0.44	3.88 ± 0.04	14.43 ± 0.21

**Table 2:** Antioxidant activities of PPPE and PAPE. Results are expressed as the mean ± sem of at least three independent assays of each sample. * *p* < 0.05 and *** *p* < 0.001 indicate significant differences compared to the control.

Plant Extract	Antioxidant Activities			
	DPPH (IC_50_ Values, µg/mL)	H_2_O_2_ (IC_50_ Values, µg/mL)	FRAP (mg AAE/g dw)	BCB (%)
PPPE	12.49 ± 0.60 *	19.96 ± 0.02 ***	374.83 ± 16.85 ***	86.83 ± 1.22 ***
PAPE	21.58 ± 4.44	37.06 ± 0.05	189.83 ± 5.29	55.64 ± 1.14

## Data Availability

Data are contained within the article.

## Supplementary Materials

The following are available online at https://www.mdpi.com/article/10.3390/foods10092219/s1, Table S1: Regression equations, linearity range, r, LOD and LOQ of all analytes, Table S2: Comparative evaluation of polyphenolic. flavonoid and anthocyanin content of peels and aril extracts of Sefri and various pomegranate (*Punica granatum*) cultivars.
