# Supplementary material for: Antioxidant Effect of Moroccan Pomegranate (Punica granatum L. Sefri Variety) Extracts Rich in Punicalagin against the Oxidative Stress Process
Source: Foods. 2021 Sep 18;10(9):2219. doi: 10.3390/foods10092219 (PMC8469689; doi:10.3390/foods10092219)
Supplement: Supplementary file 1 [file foods-10-02219-s001.zip › foods-1269242-supplementary.pdf]

Table S1. Regression equations, linearity range, r, LOD and LOQ of all analytes.

	Regression equation	linearity range (mg/mL)	r	LOD (mg/mL)	LOQ (mg/mL)
**α Punicalagin**	*Y* = 1350.8*x* - 82435	0.1 - 1	0.9965	0.0985	0.0325
**β Punicalagin**	*Y* = 1445.2*x* - 43542	0.1 - 1	0.9995	0.0403	0.122
**Gallic acid**	*Y* = 14918x - 20485	0.06 – 0.3	0.9993	0.0133	0.0403
**Ellagic acid**	*Y*= 12135*x* - 785971	0.06 – 0.3	0.985	0.0673	0.204

Table S2. Comparative evaluation of polyphenolic. flavonoid and anthocyanin content of peels and aril extracts of Sefri and various pomegranate (Punica granatum) cultivars.

Plant Extract	Polyphenols (mg GAE/g dw)	Flavonoids (mg QE/g dw)	Total Anthocyanin (mg cy-3-glu/100g dw)	α-Punicalagin (mg/g dw)	β-Punicalagin (mg/g dw)	Gallic Acid (mg/g dw)	Ellagic Acid (mg/g dw)
**Moroccan Sefri cultivar**							
PPPE	283.86 ± 17.89	185.37 ± 3.05	102.97 ± 9.19. ns	148.95 ± 2.43	302.38 ± 7.26	5.87 ±0.08	18.85 ± 0.41
PAPE	166.90 ± 18.10	57.43 ± 0.41	81.26 ± 18.39	40.40 ± 2.67	3.03 ± 0.44	3.88 ± 0.04	14.43 ± 0.21
**Tunisian Cultivar**							[1. 2]
PPPE	87.33 – 109.79	44.83 – 56.46	63.76 – 80.90	--	--	1.09 - 1.31	0.34 – 0.36
PAPE	10.59 – 13.70	4.11- 5.75	28.15 – 48.27	--	--	0.137 – 0.205	0.22 – 0.24
**Iranian cultivar**							[3. 4]
PPPE	98.24 - 250.13	18.61 - 36.4	3.95 – 29.52 (mg Cat/g)	--	--	--	--
PAPE	11.62 – 21.03	0.84 – 2.14	1.6 – 3.89 (mg Cat/g)	--	--	--	--
**Turkish cultivar**							[5. 6]
PPPE	126.11 - 212.48	9.44 - 20.52	20 – 800 mg/L	--	--	--	--
PAPE	4.22 – 9.20	1.97 – 3.97	20 – 350 mg/L	-	--	--	--
**Italian cultivar**							[7-10]
PPPE	237.7 – 612.7	0.471 – 0.881 mmol RE/g	28.45 - 32.68 μmolC3G/g	1.3 – 183.8	0.3 – 325.1	--	0.6 – 40.1
PAPE	28.39 – 103.01 mgGAE/L	29.44 – 75.15 mg/L	58.49 – 198.06 mg CE/L	--	- 165.26	4.27 -53.2 mg/L	11.22 – 41.40 mg/L
**Spanish cultivar**							[11-14]
PPPE	- 161	6.32 – 13.27 mg RE/g	- 53.52	113 - 148	74 - 107	--	13 -16.7
PAPE	141.29 – 172.5	4.77 – 18.9 mgQE/L	34.2 – 655.8 mg/L	0.3-10.3 mg/L	1-31.4 mg/L	--	4.9-152.8 mg/L
